# Association of carotid wall shear stress measured by vector flow mapping technique with ba-PWV: a pilot study

**DOI:** 10.3389/fcvm.2023.1293106

**Published:** 2023-12-08

**Authors:** Yi Cheng, Jie Chen, Qing Zhao, Jinghan Zhang, Junyi Gao

**Affiliations:** ^1^ Department of Diagnostic Ultrasound, Beijing Anzhen Hospital, Capital Medical University, Beijing, China; ^2^ Department of Cardiovascular Medicine, Beijing Shijitan Hospital, Capital Medical University, Beijing, China

**Keywords:** vector flow mapping (VFM), wall shear stress (WSS), brachial-ankle pulse wave velocity (ba-PWV), common carotid artery (CCA), subclinical atherosclerosis

## Abstract

**Objective:**

Arterial stiffness is an important tissue biomarker of the progression of atherosclerotic diseases. Brachial-ankle pulse wave velocity (ba-PWV) is a gold standard of arterial stiffness measurement widely used in Asia. Changes in vascular wall shear stress (WSS) lead to artery wall remodeling, which could give rise to an increase in arterial stiffness. The study aimed to explore the association between ba-PWV and common carotid artery (CCA) WSS measured by a newly invented vascular vector flow mapping (VFM) technique.

**Methods:**

We included 94 subjects free of apparent cardiovascular disease (CVD) and divided them into a subclinical atherosclerosis (SA) group (*N* = 47) and non subclinical atherosclerosis (NSA) group (*N* = 47). CCA WSS was measured using the VFM technique. Bivariate correlations between CCA WSS and other factors were assessed with Pearson's, Spearman's, or Kendall's coefficient of correlation, as appropriate. Partial correlation analysis was conducted to examine the influence of age and sex. Multiple linear stepwise regression was used for the analysis of independent determinants of CCA WSS. Receiver operating characteristic (ROC) analysis was performed to find the association between CCA WSS and 10-year CVD risk.

**Results:**

The overall subjects had a mean age of 47.9 ± 11.2 years, and males accounted for 52.1%. Average systolic CCA WSS was significantly correlated with ba-PWV (*r* = −0.618, *p *< 0.001) in the SA group. Multiple linear stepwise regression analysis confirmed that ba-PWV was an independent determinant of average systolic CCA WSS (*β *= −0.361, *p *= 0.003). The area under the curve (AUC) of average systolic CCA WSS for 10-year CVD risk ≥10% was 0.848 (*p *< 0.001) in the SA group.

**Conclusions:**

Average systolic CCA WSS was significantly correlated with ba-PWV and was associated with 10-year CVD risk ≥10% in the SA group. Therefore, CCA WSS measured by the VFM technique could be used for monitoring and screening subjects with potential CVD risks.

## Introduction

Arterial stiffening is an adverse structural and functional change in the artery wall (1). It has been shown to play an important role in the progression of atherosclerotic diseases and is an independent predictor of cardiovascular risk and mortality (2, 3).

Pulse wave velocity (PWV) is a clinical practice widely accepted as the gold standard of arterial stiffness measurement (4). Brachial-ankle pulse wave velocity (ba-PWV) is the most frequently applied PWV measurement in Asia, and it could be a representative of arterial stiffness for the entirety of the central and peripheral arterial system (5, 6). Ba-PWV is widely used in clinical work and epidemiological studies for its monitoring role and predicting value (7, 8). However, there are also temporal fluctuations in PWV measurements, and the estimated rates of arterial stiffening progression may deviate from the actual situation (9).

Wall shear stress (WSS) is a force exerted on the vessel wall by the blood flow (10). Changes in hemodynamics reflected by WSS promote adaptive structural remodeling of the artery wall through endothelial mechanotransduction. Artery wall remodeling could give rise to an increase in arterial stiffness (11). Therefore, WSS may be associated with PWV, which represents systemic arterial stiffness.

The vascular vector flow mapping (VFM) technique was first proposed in 2017. It is an application that measures carotid WSS by combining speckle tracking and Doppler imaging with two-directional echo beams (12–15). Previous study has confirmed the accuracy and feasibility of this technique (14). The study aimed to explore the correlation between ba-PWV and the common carotid artery (CCA) WSS measured by the VFM technique and to explore the association between CCA WSS and 10-year cardiovascular disease (CVD) risk.

## Materials and methods

### Patients

We examined subjects (*N* = 579) who visited the Department of Diagnostic Ultrasound and Health Examination Center at Beijing Anzhen Hospital for carotid artery ultrasound examination from October 2022 to December 2022. Subjects with previous CVD were excluded (*N* = 402). CVD was defined as previously diagnosed coronary artery disease, cerebrovascular disease, peripheral vascular disease, heart failure, rheumatic heart disease, congenital heart disease, cardiomyopathies, and severe cardiac arrhythmia such as atrial fibrillation (AF) (16).

Subjects with newly discovered carotid artery stenosis during enrollment were excluded (*N* = 8) (17). Subjects who refused to take VFM examination were excluded (*N* = 2). Subjects who did not participate in ba-PWV examinations were excluded (*N* = 73). We finally enrolled 94 subjects for analysis. All subjects included received routine physical examinations, medical history collection, blood biochemical examinations, regular ultrasounds, and VFM examinations. Ba-PWV and 24-hour ambulatory blood pressure monitoring were also performed. Average Ba-PWV combining bilateral values was used for data analysis. The China-PAR score was used to present the 10-year CVD risk of the subjects. Risk classification criteria were defined as: low risk (<5%), medium risk (5%–9.9%), or high risk (≥10%) (18).

**Figure 1:**
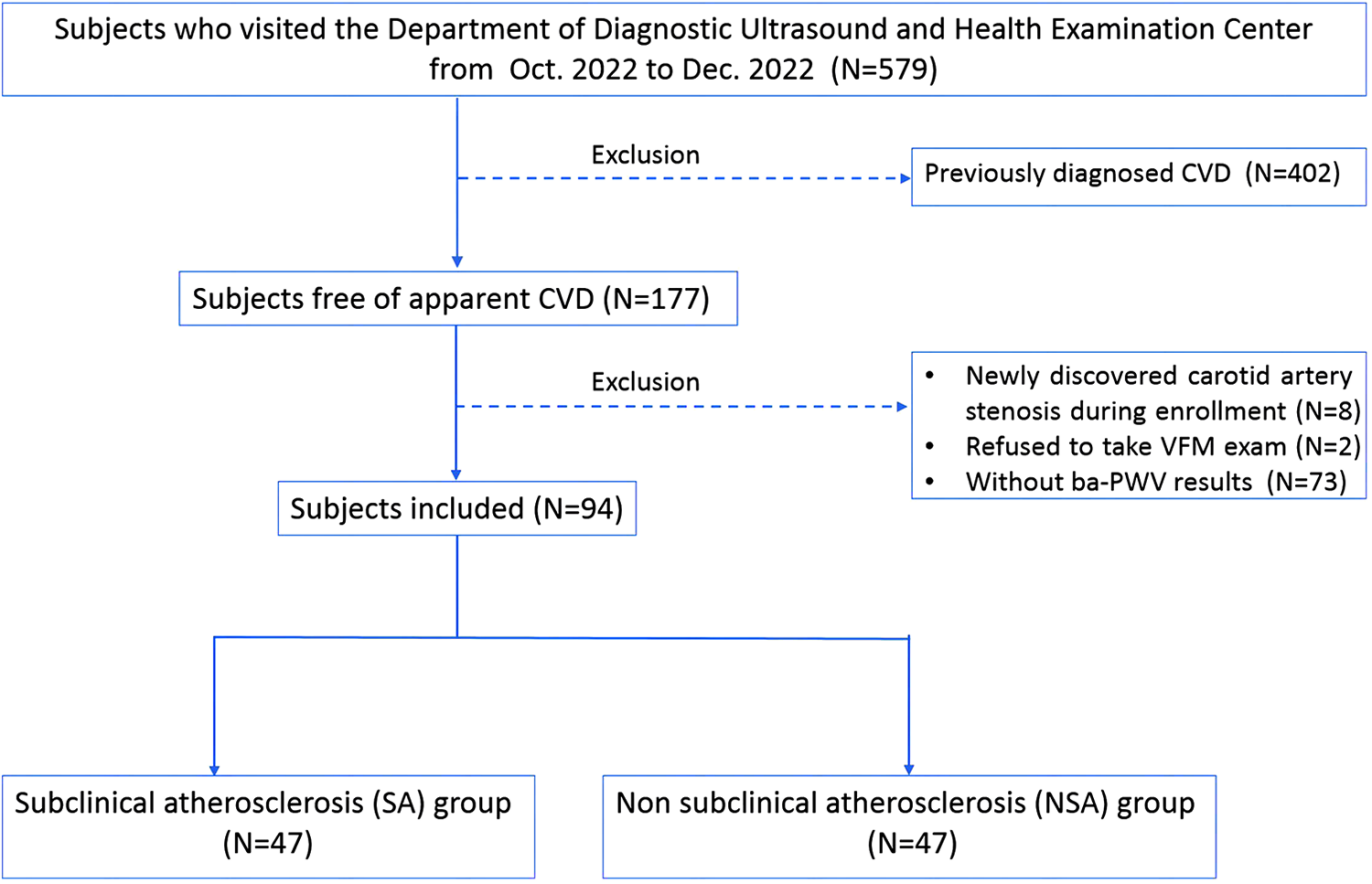
Flow chart of the study. CVD, cardiovascular diseases; CCA, common carotid artery; WSS, wall shear stress; VFM, vector flow mapping; Ba-PWV, brachial-ankle pulse wave velocity. CVD was defined as coronary artery disease, cerebrovascular disease, peripheral vascular disease, heart failure, rheumatic heart disease, congenital heart disease, cardiomyopathies, and severe cardiac arrhythmias such as atrial fibrillation.

The flow chart is shown in Figure 1.

### Regular ultrasound

Subjects took a rest for at least 30 min before they underwent carotid artery examination. Carotid artery ultrasound was performed by two experienced ultrasound clinicians using LISENDO880LE (Hitachi, Tokyo, Japan) with a linear probe (L441, Hitachi, Tokyo, Japan).

The presence of carotid artery plaque was evaluated before the VFM examination (19). The posterior wall of the distal segment (1 cm proximal to the bifurcation) of CCA without plaque was chosen for mean intima-media thickness (IMT) measurement. Unilateral CCA IMT was calculated automatically tracing the luminal edge of the high-echoic layer of the intima. Unilateral CCA peak systolic velocity (PSV), end diastolic velocity (EDV), resistive index (RI), and pulsate index (PI) were calculated automatically. Unilateral systo-diastolic index (SDI) was calculated as PSV/EDV. Average CCA IMT and flow parameters were measured by combining bilateral data.

### VFM examination

**Figure 2:**
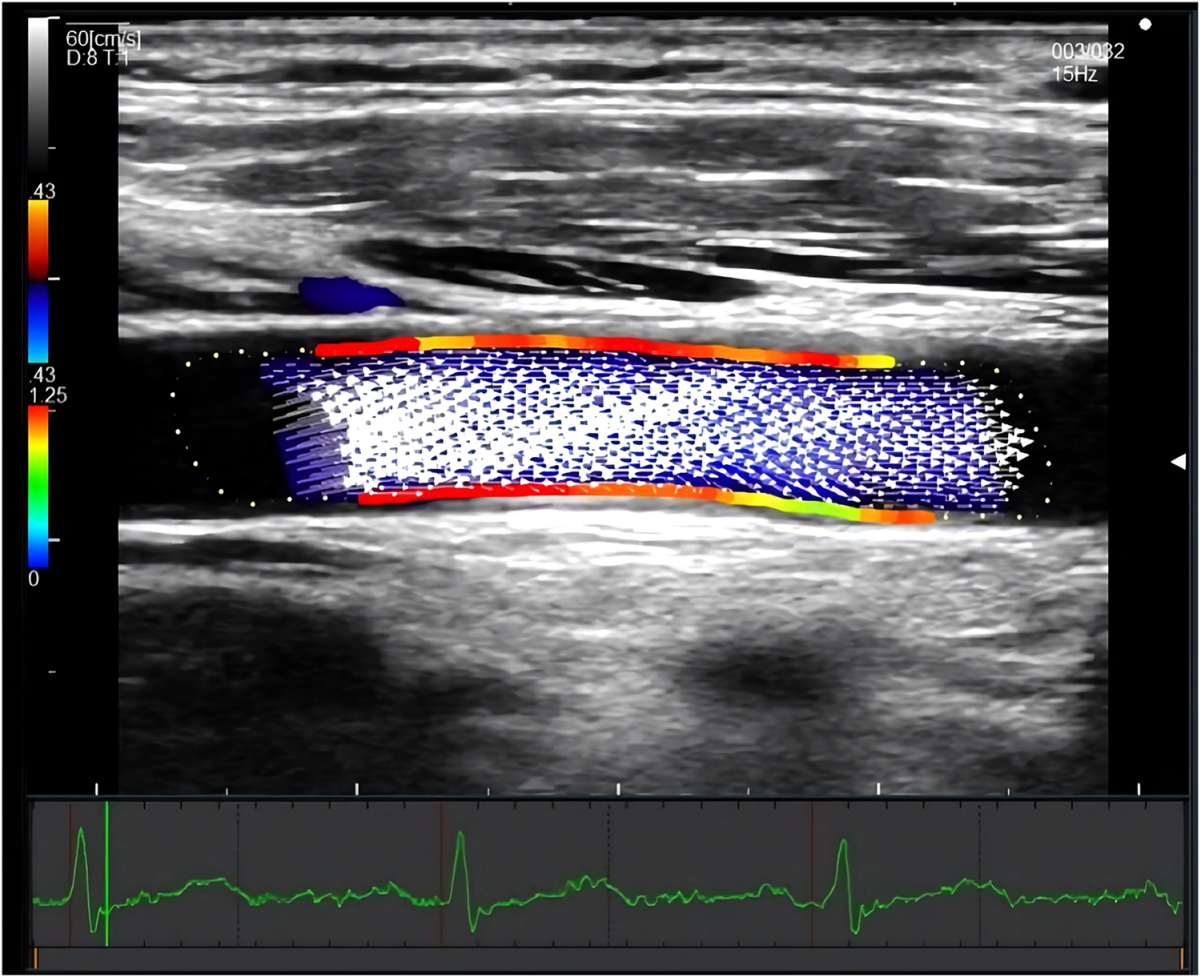
Measurement of systolic CCA WSS by VFM technique. The figure showed a longitudinal section of the common carotid artery. The red area indicated a relatively high wall shear stress, and the green area indicated a relatively low wall shear stress. The white arrow represents the blood flow velocity vector of the carotid artery. The electrocardiogram on the bottom ensured the time of measurement.

CCA WSS was measured at 1 cm proximal to the bifurcation without plaque in the supine position. If plaque existed at the proximal bifurcation segment, the middle segment of CCA was examined instead. Images of three heartbeats were saved as raw data in each measurement. The depth was controlled to less than 3.5 cm. The steering angle of the color Doppler flow was adjusted from 0 to 30 degrees. The dynamic range and velocity range were kept as small as possible within the aliasing correction, and the gain was adjusted as necessary. The crossbeam was automatically set and was adjusted by 5-degree increments to maximize the crossbeam Doppler signal in the vessel. The unilateral systolic CCA WSS value of the anterior and posterior walls was calculated automatically using DAS-RS1 software (Hitachi, Tokyo, Japan). (Shown in Figure 2) Average systolic WSS was measured by combining the values of bilateral CCA WSS.

### Grouping method

Subjects were finally divided into two groups. Subjects with average CCA IMT ≥ 0.90 mm or with carotid atherosclerotic plaque were assigned to the subclinical atherosclerosis (SA) group (*N* = 47). Subjects without carotid atherosclerotic plaque and with average CCA IMT < 0.90 mm were assigned to the non subclinical atherosclerosis (NSA) group (*N* = 47) (20–22).

### Statistical analysis

Continuous variables were reported as mean ± standard deviation (SD) or median with interquartile range as appropriate. Categorical variables were reported as numbers and percentages. Differences between groups were compared using the Student's *t*-test or Mann–Whitney *U*-test, as appropriate. Bivariate correlations between CCA WSS and other variables were assessed with Pearson's, Spearman's, or Kendall's coefficient of correlation as appropriate. Partial correlation analysis was used to examine the influence of age and sex. Multiple linear stepwise regression was used for analysis of independent determinants of average systolic CCA WSS. Receiver operating characteristic (ROC) analysis was used to explore the association of CCA WSS and ba-PWV with 10-year CVD risk. All tests of significance were two-tailed, and a *p-value* ≤ 0.05 was considered statistically significant. Analyses were performed using SPSS 26.0 (SPSS, Chicago, IL, USA). G power was calculated using the software G Power 3.1.9.7.

## Results

### Basic characteristics

The overall subjects had a mean age of 47.9 ± 11.2 years, and males accounted for 52.1%. Compared with the NSA group (*N* = 47), subjects in the SA group (*N* = 47) were older (53.0 ± 11.0 years vs. 42.8 ± 8.9 years, *p *< 0.001). The two groups had a comparable incidence of hypertension (HTN) history, incidence of diabetes mellitus (DM) history, body mass index (BMI), waist circumference (WC), 24-hour average systolic blood pressure (SBP), 24-hour average heart rate (HR), TG levels, TCHO levels, LDL-C levels, and HbA1C levels. We found that 24-hour average diastolic blood pressure (DBP) was lower in the SA group (80.2 ± 9.9 mmHg vs. 84.8 ± 11.6 mmHg, *p *= 0.041), and average CCA IMT was higher in the SA group (0.679 ± 0.146 mm vs. 0.608 ± 0.083 mm, *p *= 0.005).

In the SA group, average systolic CCA WSS was lower (1.049 ± 0.295 Pa vs. 1.130 ± 0.361 Pa, *p *= 0.242) and average ba-PWV was higher (1,563 ± 327 cm/s vs. 1,543 ± 217 cm/s, *p *= 0.731), but the differences were not significant. The SA group had a higher China-PAR score compared with the NSA group [8.5 (4.3, 12.3) vs. 3.8 (2.1, 8.4), *p *= 0.005].

Basic characteristics of patients are shown in Table 1.

**Table 1:** Basic characteristics of the subjects.

	All (*N* = 94)	SA (*N* = 47)	NSA (*N* = 47)	*p* value
Age (years)	47.9 ± 11.2	53.0 ± 11.0	42.8 ± 8.9	<0.001
Male (%)	49 (52.1%)	24 (51.1%)	25 (53.2%)	0.836
HTN history (%)	80 (85.1%)	42 (89.4%)	38 (80.9%)	0.247
DM history (%)	23 (24.5%)	15 (31.9%)	8 (17.0%)	0.093
Smoking history (%)	34 (36.2%)	21 (44.7%)	13 (27.7%)	0.086
BMI (kg/m^2^)	27.5 ± 4.8	26.6 ± 4.1	28.4 ± 5.2	0.067
WC (cm)	96.7 ± 12.1	95.9 ± 10.2	97.6 ± 13.7	0.481
24-hour average SBP (mmHg)	129.2 ± 13.9	127.0 ± 11.9	131.5 ± 15.5	0.117
24-hour average DBP (mmHg)	82.5 ± 11.0	80.2 ± 9.9	84.8 ± 11.6	0.041
24-hour variation of SBP (%)	11.1 ± 3.0	11.0 ± 2.6	11.2 ± 3.3	0.767
24-hour variation of DBP (%)	11.9 ± 6.0	12.2 ± 8.2	11.5 ± 2.2	0.588
24-hour average HR (bpm)	71.4 ± 10.2	69.6 ± 9.8	73.2 ± 10.2	0.085
UA (μmol/L)	353.4 ± 100.7	352.6 ± 112.1	354.2 ± 89.0	0.939
TG (mmol/L)	1.81 (1.21, 2.44)	1.90 (1.26, 2.53)	1.70 (1.12, 2.37)	0.360
TCHO (mmol/L)	5.24 ± 1.00	5.36 ± 0.97	5.12 ± 1.03	0.254
HDL-C (mmol/L)	1.22 ± 0.29	1.29 ± 0.33	1.16 ± 0.23	0.023
LDL-C (mmol/L)	3.02 ± 0.81	3.05 ± 0.82	2.99 ± 0.81	0.691
hs-CRP (mg/L)	0.97 (0.59, 2.46)	0.87 (0.57, 1.79)	1.21 (0.67, 3.29)	0.246
HbA1c (%)	6.0 ± 0.8	6.1 ± 0.8	5.9 ± 0.7	0.176
Average CCA IMT (mm)	0.643 ± 0.123	0.679 ± 0.146	0.608 ± 0.083	0.005
Average systolic CCA WSS (Pa)	1.090 ± 0.330	1.049 ± 0.295	1.130 ± 0.361	0.242
Average CCA PSV (cm/s)	74.1 ± 15.4	70.8 ± 14.6	77.5 ± 15.6	0.037
Average CCA EDV (cm/s)	19.4 ± 4.1	18.9 ± 4.3	20.0 ± 3.8	0.231
Average CCA RI	0.74 ± 0.07	0.729 ± 0.048	0.756 ± 0.089	0.074
Average CCA PI	1.67 ± 0.32	1.62 ± 0.28	1.72 ± 0.35	0.141
Average CCA SDI	3.90 ± 0.68	3.84 ± 0.67	3.95 ± 0.70	0.442
Average ba-PWV (cm/s)	1,553 ± 277	1,563 ± 327	1,543 ± 217	0.731
China-PAR 10-year CVD score (%)	6.0 (3.1, 10.8)	8.5 (4.3, 12.3)	3.8 (2.1, 8.4)	0.005
High 10-year CVD risk (%)	25 (26.6%)	16 (34.0%)	9 (19.1%)	0.102

SA, subclinical atherosclerosis; NSA, non-subclinical atherosclerosis; HTN, hypertension; DM, diabetes mellitus; BMI, body mass index; WC, Waist circumference; SBP, systolic blood pressure; DBP, diastolic blood pressure; HR, heart rate; bpm, beat per minute; UA, uric acid; TG, triglycerides; TCHO, total cholesterol; HDL-C, high-density lipoprotein cholesterol; LDL-C, low-density lipoprotein cholesterol; hs-CRP, high-sensitivity C reactive protein; HbA1c, glycated hemoglobin; CCA, common carotid artery; WSS, wall shear stress; IMT, inter-medium thickness; Ba-PWV, brachial-ankle pulse wave velocity; PSV, peak systolic velocity; EDV, end diastolic velocity; RI, resistive index; PI, pulsate index; SDI, systo-diastolic index; China-PAR score, prediction for atherosclerotic cardiovascular disease risk score in China.

### Correlation analysis

In the SA group, average systolic CCA WSS was significantly correlated with average ba-PWV (*r* = −0.618, *p *< 0.001), age (*r* = −0.626, *p *< 0.001), average CCA IMT (*r* = −0.479, *p *= 0.001), average CCA PSV (*r* = 0.629, *p *< 0.001), and average CCA EDV (*r* = 0.642, *p *< 0.001). The significance of correlations between CCA WSS and ba-PWV, CCA PSV, and CCA EDV remained after age and sex were adjusted. The significance of the correlation between CCA WSS and IMT weakened after age and sex were adjusted. In the NSA group, average systolic CCA WSS was correlated with CCA PSV (*r* = 0.488, *p *= 0.001). No significant correlation was observed between CCA WSS and average ba-PWV. China-PAR score had significant correlations with CCA WSS in both the SA group (*r* = −0.632, *p *< 0.001) and the NSA group (*r* = −0.400, *p = *0.005). Results from correlation analysis were shown in Supplementary Table S1.

Age, average CCA IMT, CCA PSV, CCA EDV, and ba-PWV were included in multiple linear stepwise regression analyses for determinants of average systolic CCA WSS. Average CCA PSV (*β *= 0.452, *p *= 0.001) and ba-PWV (*β *= −0.361, *p *= 0.003) were significant and independent determinants of CCA WSS. Results from multiple linear stepwise regression analysis were shown in Supplementary Table S2.

### ROC analysis

**Figure 3:**
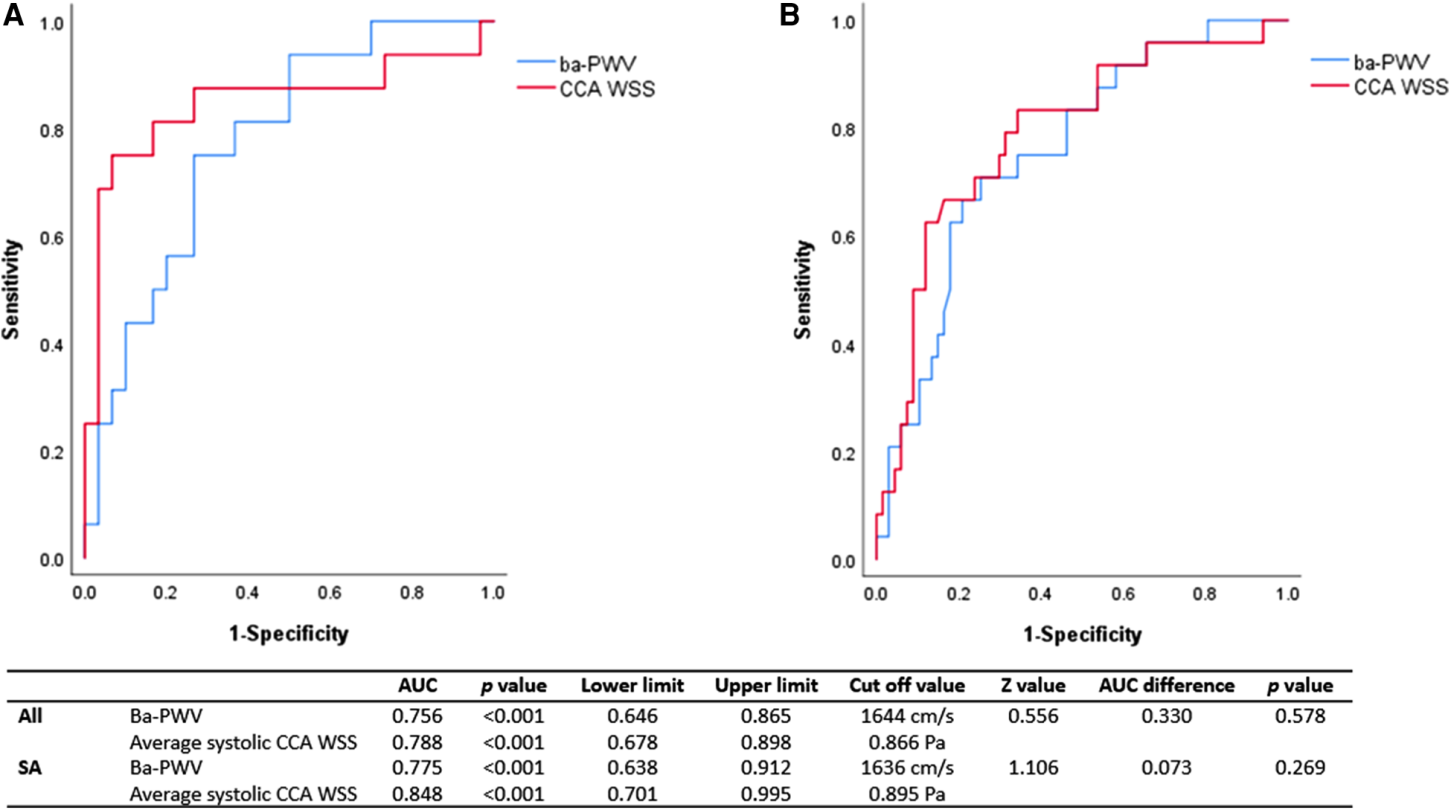
ROC analysis of CCA WSS and ba-PWV for 10-year CVD risk ≥10%. ROC, receiver operating characteristic; AUC, area under the curve; Ba-PWV, brachial-ankle pulse wave velocity; CCA, common carotid artery; WSS, wall shear stress; SA, subclinical atherosclerosis. The AUC of CCA WSS for high risk of 10-year CVD was 0.848 (*p *< 0.001) in SA subjects (**A**) and 0.788 (*p *< 0.001) in the overall subjects (**B**). The AUC of CCA WSS was comparable with that of ba-PWV in SA and the overall subjects. The cutoff value of CCA WSS was 0.866 Pa in the overall subjects and 0.895 Pa in the SA subjects.

ROC analysis was conducted in SA subjects and in the overall subjects to represent the association between CCA WSS (as well as ba-PWV) and 10-year CVD risk. The area under the curve (AUC) of CCA WSS and ba-PWV were 0.788 (*p *< 0.001) and 0.756 (*p *< 0.001) for high 10-year CVD risk (≥10%) in the overall subjects. In the SA group, the AUC of CCA WSS and ba-PWV was 0.848 (*p *< 0.001) and 0.775 (*p *< 0.001), respectively. Although no significant difference could be found, the trend still indicated that CCA WSS had at least comparable AUC with ba-PWV. Results from ROC analysis are shown in Figure 3.

## Discussion

Our data showed that the average systolic CCA WSS value was 1.090 ± 0.330 Pa in the subjects free of apparent CVD, and our result was consistent with He's research in 2022. They reported an average CCA WSS of 1.28 ± 0.33 Pa in a population without obvious CVD (23). Adel. M. and colleagues elucidated that WSS higher than 1.5 Pa indicates a healthy artery wall, while WSS lower than 0.4 Pa promotes the development of arteriosclerosis (24). In our study, the average systolic CCA WSS was between normal and atherosclerosis tendency conditions. Considering the characteristics of the patients, our result was reasonable.

Our study is the first known to propose a significant correlation between ba-PWV and CCA WSS measured by the VFM technique in a subclinical atherosclerosis population. Multiple linear stepwise regression analysis confirmed that ba-PWV was an independent determinant of average systolic CCA WSS. Previous research indicated that WSS is a “local” factor for arterial remodeling (25), and endothelial dysfunction in different artery beds may present heterogeneity in an individual. The significant correlation between CCA WSS and ba-PWV (represents systemic arterial stiffness) showed a possibility that CCA WSS might not only be a regional parameter but could also reflect a “systemic” characteristic.

There are certain mechanisms underlying the association between CCA WSS and ba-PWV, including endothelial dysfunction, extracellular matrix degeneration, and inflammation (26). Endothelial dysfunction might be an important bridge linking CCA WSS and ba-PWV. Changes in mechanical forces (such as WSS) in the artery microenvironment affect endothelial functions by influencing the phenotype and function of endothelial cells. Alternation of the endothelium to a dysfunctional status leads to the pathogenesis of cardiovascular diseases such as atherosclerosis (27). On the other hand, changes in endothelial function often involve structural alternation of the artery wall, which could lead to an increase in arterial stiffness (3). In addition, it has been reported that remodeling of extracellular matrix protein, which could be induced by changes in WSS, may also have a role in arterial stiffening (28, 29).

Therefore, CCA WSS and ba-PWV are two indicators that gradually change together on the road from “healthy” to “arteriosclerosis status”. We speculate the reason that the correlation was more prominent in the SA group might be more obvious structural and functional alterations in this group. However, we could not rule out the possible association between ba-PWV and CCA WSS in the NSA group, and future research is needed in other populations as well.

In our study, carotid hemodynamic parameters, including CCA PSV and CCA EDV, represented significant correlations with CCA WSS. As an important determinant of WSS, blood velocity promotes adaptive structural remodeling of the artery wall through endothelial mechanotransduction (30). Previous studies revealed that age has a certain impact on vascular hemodynamic parameters. Thus, we speculate that the influence of age on the progress of atherosclerosis might partially be mediated by blood flow parameters, which should be paid more attention to in future studies (31).

We are also the first known to propose the significant association between CCA WSS measured by VFM technique and a high risk of 10-year CVD (China-PAR score ≥10%). In addition, the AUC of CCA WSS was comparable with that of ba-PWV.

Arterial stiffness has been shown to play an important role in the progression of atherosclerotic diseases. As a representative of systemic arterial stiffness, ba-PWV is an independent predictor of cardiovascular risk and mortality (2, 3). CCA WSS reflects changes in endothelial function, which is the accumulation of the impact of various risk factors on the arterial wall. It also reflects an interaction between genetic background and environmental impact (comorbidities, life habits, etc.). Therefore, endothelial function might be a potential representative of the overall CVD risk of an individual (32). In our data, we found a significant correlation between CCA WSS and China-PAR score in both the SA and NSA groups. So we speculate that CCA WSS might also be associated with CVD risk. The disease burdens of CVD including coronary heart disease and stroke are increasing and contribute to the major challenge of public health policy (33). There are different tools for CVD risk evaluation that guide public health and clinical practice in different regions (34, 35). We chose the most suitable evaluation system for our study. The China-PAR project proposed validated equations predicting 10-year CVD risk in an asymptomatic population. A clear relationship was observed between China-PAR scores and the incidence of 10-year CVD (18).

Our data showed that the AUC of CCA WSS for high risk of 10-year CVD was 0.848 (*p *< 0.001) in SA subjects and 0.788 (*p *< 0.001) in the overall subjects. The AUC of CCA WSS was comparable with that of ba-PWV (shown in Figure 3). In addition, the AUC of CCA WSS in the SA group seemed to be better than the AUC of the overall subjects. These results confirmed our speculation that CCA WSS may correlate with CVD risk and a greater extent of endothelial dysfunction as well as other pathological changes that may exist in the SA group.

We believe our result may bear some clinical value. Ba-PWV is widely used in clinical works and epidemiological studies for its monitoring role in the progression of atherosclerotic diseases and predicting value on CVD prognosis (7, 8). It is crucial to monitor ba-PWV regularly for subjects with potential cardiovascular risks. However, measurements of ba-PWV may depend on the device types used. Not only that, the temporal fluctuations of PWV measurements lead to the deviation of the estimated arterial stiffening rates from the actual situation (9, 36). Although CCA WSS could be measured using various methods such as computed flow dynamics (CFD) and four-dimensional magnetic resonance imaging (MRI), they may not be suitable for daily clinical practice (37, 38). Carotid ultrasound is a commonly used monitoring method and the measurement of CCA WSS using VFM technique bears the advantages of demanding little time and being non-invasive and cheap. It is quite convenient to measure CCA WSS simultaneously with a regular carotid ultrasound. Therefore, CCA WSS measured by the VFM technique could be used for large-scale screening of populations with potential risks before regular ba-PWV is needed and for monitoring during the interval of ba-PWV measurements.

This research has some limitations.

First, we only included subjects free of apparent CVD with a relative young age, and the association between CCA WSS and ba-PWV was only significant in subclinical atherosclerosis subjects. We could not rule out the possible association between them in healthy subjects or other populations. Additionally, this does not mean that CCA WSS could replace ba-PWV. Further studies in other populations are needed to expand the value of CCA WSS. The second limitation was that our sample size was limited. However, we used the software G Power 3.1.9.7 to calculate the power of bilateral correlation (0.8) and the power of multiple linear regression analysis (0.73), which were acceptable. We also used partial correlation analysis to evaluate the influence of age and sex.

## Conclusion

We measured average systolic CCA WSS using the newly invented VFM technique. We are the first known to propose that average systolic CCA WSS was significantly correlated with ba-PWV in a subclinical atherosclerosis population. Multiple stepwise regression analysis confirmed ba-PWV was significantly and independently associated with average systolic CCA WSS. In addition, CCA WSS had a comparable AUC with ba-PWV for high 10-year CVD risk (China-PAR score ≥10%). Therefore, CCA WSS measured by the VFM technique might be used for screening and monitoring of subjects with potential CVD risks.

## Data Availability

The raw data supporting the conclusions of this article will be made available by the authors, without undue reservation.
