# Supplementary material for: Association of carotid wall shear stress measured by vector flow mapping technique with ba-PWV: a pilot study
Source: Front Cardiovasc Med. 2023 Dec 8;10:1293106. doi: 10.3389/fcvm.2023.1293106 (PMC10748391; doi:10.3389/fcvm.2023.1293106)
Supplement: Supplementary file 1 [file Table1.docx]

**Supplementary Table 1 Correlations between average systolic CCA WSS and parameters.**

|  | **SA group** | | **Age adjusted** | | **Age and sex adjusted** | | **NSA group** | |
| --- | --- | --- | --- | --- | --- | --- | --- | --- |
|  | **r** | ***p* value** | **r** | ***p* value** | **r** | ***p* value** | **r** | ***p* value** |
| Age (years) | -0.626** | <0.001 | -- | -- | -- | -- | 0.090 | 0.547 |
| Male (%) | 0.104 | 0.395 | 0.031 | 0.840 | -- | -- | 0.150 | 0.216 |
| Smoking history (%) | 0.310 | 0.130 | 0.220 | 0.142 | 0.287 | 0.056 | -0.017 | 0.887 |
| BMI (kg/m^2^) | -0.115 | 0.442 | -0.468** | 0.028 | -0.490** | 0.001 | -0.515** | 0.000 |
| WC (cm) | -0.193 | 0.195 | -0.430** | 0.023 | -0.518** | 0.001 | -0.369** | 0.011 |
| 24-hour average SBP (mmHg) | -0.143 | 0.337 | -0.101 | 0.230 | -0.093 | 0.584 | -0.361* | 0.013 |
| 24-hour average DBP (mmHg) | 0.070 | 0.642 | -0.084 | 0.457 | -0.042 | 0.806 | -0.426** | 0.003 |
| 24-hour variation SBP (%) | -0.143 | 0.385 | 0.204 | 0.227 | 0.211 | 0.211 | -0.120 | 0.472 |
| 24-hour variation DBP (%) | -0.017 | 0.920 | -0.064 | 0.715 | -0.077 | 0.652 | -0.041 | 0.807 |
| 24-hour average HR (bpm) | 0.221 | 0.201 | 0.261 | 0.319 | 0.273 | 0.101 | -0.186 | 0.169 |
| UA (μmol/L) | -0.239 | 0.106 | -0.428** | 0.009 | -0.506** | 0.001 | -0.001 | 0.997 |
| TG (mmol/L) | -0.054 | 0.717 | -0.087 | 0.307 | -0.092 | 0.574 | -0.085 | 0.568 |
| TCHO (mmol/L) | 0.015 | 0.920 | 0.011 | 0.468 | 0.027 | 0.867 | -0.081 | 0.587 |
| HDL-C (mmol/L) | -0.003 | 0.982 | 0.198 | 0.080 | 0.228 | 0.156 | 0.057 | 0.705 |
| LDL-C (mmol/L) | 0.127 | 0.396 | 0.042 | 0.521 | 0.057 | 0.729 | -0.063 | 0.673 |
| hs-CRP (mg/L) | -0.042 | 0.783 | -0.232 | 0.168 | -0.228 | 0.158 | -0.270 | 0.066 |
| HbA1c (%) | -0.159 | 0.290 | -0.083 | 0.717 | -0.075 | 0.647 | -0.268 | 0.069 |
| Average ba-PWV (cm/s) | -0.618** | <0.001 | -0.408** | 0.019 | -0.405** | 0.010 | -0.291 | 0.053 |
| Average CCA IMT (mm) | -0.479** | 0.001 | -0.301 | 0.181 | -0.308 | 0.053 | -0.086 | 0.565 |
| Average CCA PSV | 0.629** | <0.001 | 0.542** | 0.001 | 0.540** | <0.001 | 0.488** | 0.001 |
| Average CCA EDV | 0.642** | <0.001 | 0.467** | 0.006 | 0.464** | <0.001 | 0.210 | 0.170 |
| Average CCA RI | -0.043 | 0.780 | 0.190 | 0.267 | 0.184 | 0.257 | 0.051 | 0.740 |
| Average CCA PI | -0.003 | 0.985 | 0.100 | 0.622 | 0.086 | 0.600 | 0.227 | 0.138 |
| Average CCA SDI | -0.107 | 0.485 | 0.140 | 0.398 | 0.135 | 0.405 | 0.339* | 0.024 |
| China-PAR 10-year CVD score (%) | -0.632** | <0.001 | -0.293* | 0.048 | -0.293* | 0.050 | -0.400** | 0.005 |

**Abbreviations:** SA: subclinical atherosclerosis; NSA: non subclinical atherosclerosis; BMI: body mass index; WC: Waist circumference; SBP: systolic blood pressure; DBP: diastolic blood pressure; HR: heart rate; UA: uric acid; TG: triglycerides; TCHO: total cholesterol; HDL-C: high density lipoprotein cholesterol; LDL-C: low density lipoprotein cholesterol; hs-CRP: high-sensitivity C reactive protein; HbA1c: glycated haemoglobin; CCA: common carotid artery; WSS: wall shear stress; IMT: inter-medium thickness; Ba-PWV: brachial-ankle pulse wave velocity; PSV: peak systolic velocity; EDV: end diastolic velocity; RI: resistive index; PI: pulsate index; SDI: systo-diastolic index; China-PAR score: prediction for atherosclerotic cardiovascular disease risk score in China. “**” represents a p value < 0.01 and “*” represents a p value < 0.05.

**Supplementary Table 2 Independent determinant of systolic CCA WSS from multiple stepwise regression analysis in SA group.**

|  | **β** | **t** | ***p* value** | **Adjusted R^2^** | ***p* value** |
| --- | --- | --- | --- | --- | --- |
| Constant |  | 3.939 | <0.001 | 0.684 | 0.000 |
| Age | -0.085 | -0.672 | 0.506 |  |  |
| Average Ba-PWV | -0.361 | -3.228 | 0.003 |  |  |
| Average CCA IMT | -0.149 | -1.475 | 0.148 |  |  |
| Average CCA PSV | 0.452 | 3.713 | 0.001 |  |  |
| Average CCA EDV | 0.113 | 0.825 | 0.414 |  |  |

**Abbreviations:** CCA: common carotid artery; WSS: wall shear stress; IMT: inter-medium thickness; Ba-PWV: brachial-ankle pulse wave velocity; PSV: peak systolic velocity; EDV: end diastolic velocity.
